# ScLinear predicts protein abundance at single-cell resolution

**DOI:** 10.1038/s42003-024-05958-4

**Published:** 2024-03-04

**Authors:** Daniel Hanhart, Federico Gossi, Maria Anna Rapsomaniki, Marianna Kruithof-de Julio, Panagiotis Chouvardas

**Affiliations:** 1 Urology Research Laboratory, Department for BioMedical Research, University of Bern, 3008 Bern, Switzerland; 2 IBM Research Europe, Säumerstrasse 4, 8803 Rüschlikon, Switzerland; 3 Department of Urology, Inselspital, Bern University Hospital, University of Bern, 3010 Bern, Switzerland

**Keywords:** Machine learning, Software, Transcriptomics

## Abstract

Single-cell multi-omics have transformed biomedical research and present exciting machine learning opportunities. We present scLinear, a linear regression-based approach that predicts single-cell protein abundance based on RNA expression. ScLinear is vastly more efficient than state-of-the-art methodologies, without compromising its accuracy. ScLinear is interpretable and accurately generalizes in unseen single-cell and spatial transcriptomics data. Importantly, we offer a critical view in using complex algorithms ignoring simpler, faster, and more efficient approaches.

## Abstract (web summary)

scLinear is a simple linear regression model that outperforms complex machine/deep learning approaches in predicting protein abundance at single-cell resolution.

## Introduction

The use of new single-cell profiling technologies has revolutionized our comprehension of complex biological systems. Single-cell RNA sequencing (scRNA-seq) has allowed the generation of expression landscapes across cell types, tissues or organisms with unprecedented resolution^1,2^. More recently, single-cell multi-omics have enabled the simultaneous measurement of different combinations of (epi)genomic, transcriptomic and proteomic profiles in the same cell. Notable examples include cellular indexing of transcriptomes and epitopes by sequencing (CITE-seq) that quantifies paired RNA and surface protein levels^3^, and methods that integrate the assay for transposase-accessible chromatin (ATAC) for chromatin accessibility with single-nucleus RNA sequencing^4^. These datasets offer immense biological value and present exciting opportunities for machine learning applications in the biomedical field. The two main tasks arising from such approaches are modality matching (i.e. aligning single modalities) and modality prediction (i.e. inferring one modality from another). Those tasks have been the focus of multiple studies^5–9^ and the main objectives of a recent crowdsourcing competition, where the winning methods utilized Deep Neural Networks and Kernel Ridge Regression (KRR), respectively^10^. As documented above, complex machine- and deep-learning (ML/DL) models are dominating research in the single-cell field. Although the potential of ML/DL-based single-cell methods is undisputed, this trend may lead to overshadowing simpler and more interpretable algorithms that could potentially be equally or more accurate and effective. In our study, we tested this hypothesis for the task of modality prediction in single-cell multi-omics.

## Results

### ScLinear outperforms complex machine/deep learning models

**Fig. 1:**
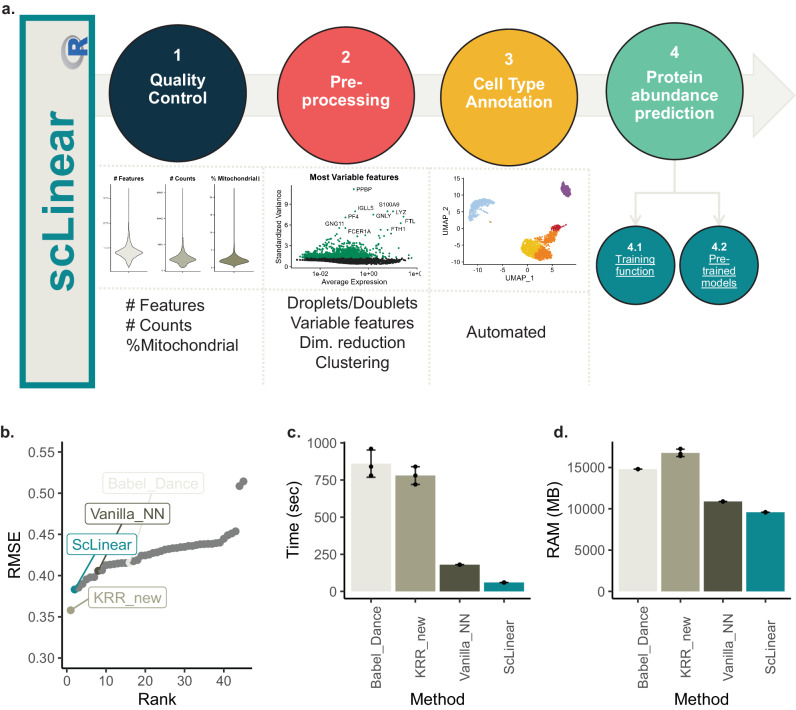
ScLinear overview, efficiency, and accuracy. **a** ScLinear schematic. **b** RMSE of predicted values in the NeurIPS test set. **c** Training time; *n* = 3 random seeds. **d** Median RAM usage across training; *n* = 3 random seeds. The standard deviation of different seeds is shown in error bars.

We developed scLinear, a versatile tool that utilizes a linear regression approach to predict single-cell protein levels from gene expression. ScLinear is developed as a comprehensive end-to-end framework, including all necessary single-cell data analysis steps, namely quality control, pre-processing and cell type annotation (Fig. 1a). To ensure community reuse, scLinear offers both the option to train new models on custom data, and also comes equipped with pre-trained models that can be fine-tuned for different tasks. We utilized the NeurIPS 2021 competition modality prediction dataset, which contains 66,175 paired gene expression (GEX) to antibody-derived tags (ADT) measurements from bone marrow mononuclear cells (BMMCs) of human donors^10^. We used the train and test sets as used in the competition to evaluate the submissions. Moreover, we established an additional set of methods for comparison: a. adapted KRR, removing the dimensionality reduction of the ADT modality (KRR_new), b. Babel, one of the pioneering methods based on an autoencoder architecture^9^ as implemented in the Dance package^11^ (Babel_Dance) and c. a naive Neural Network (Vanilla_NN). To perform a fair comparison among the 4 methods, we adapted the same pre-processing for all of them. This strategy will allow us to compare to the methods participating in the competition and at the same time deepen the comparison of scLinear to different ML algorithms.

Initially, we evaluated the performance of the four methods (scLinear, KRR_new, Babel_Dance, and Vanilla_NN) in the competition test dataset by estimating the root mean squared error (RMSE) and compared their performance to all the other submissions (Fig. 1b). Strikingly, ScLinear performed at state-of-the-art levels and was only outperformed by the new KRR approach. Next, we explored the computational efficiency of the four methods. ScLinear is vastly more computationally efficient both in terms of time needed for training (Fig. 1c) and RAM usage (Fig. 1d). Briefly, scLinear is 3x faster compared to the naïve NN, 14x faster compared to the Babel_Dance and 13x faster than the new KRR approach. Importantly, it uses significantly less RAM (35% compared to Babel, 42% compared to KRR) across the training process. We repeated the same process 3 times, and the results are consistent (Fig. 1c, d).

### ScLinear’s predictions in independent datasets and in various drop-out rates

**Fig. 2:**
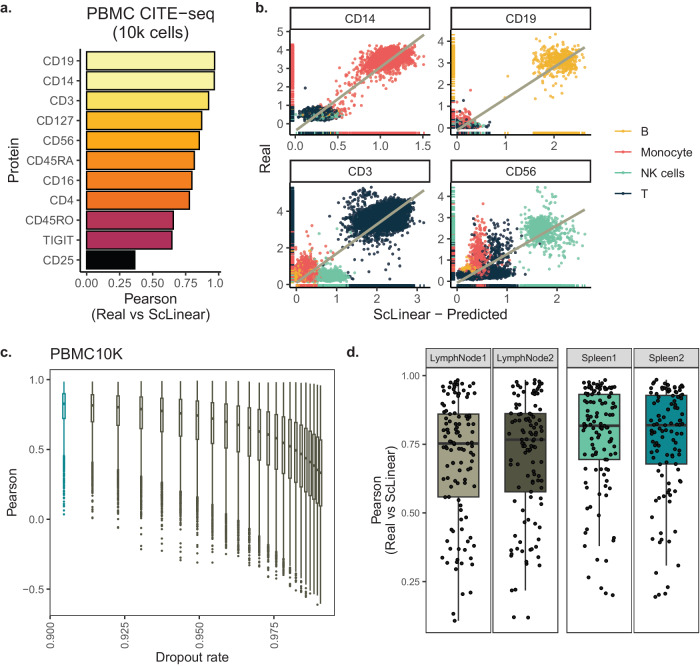
ScLinear robustness and generalizability. **a** Pearson correlation in unseen PBMC10K set split by protein. **b** ScLinear predictions in relationship to the measured data for 4 known markers colored by cell type. **c** Pearson correlation of scLinear predicted values compared to ADT across different levels of dropouts. **d** Prediction performance for mouse lymph node and spleen data set. Two-sided Pearson correlation was used.

We then sought to examine scLinear’s generalizability and robustness. We evaluated the model trained on the NeurIPS dataset on an unseen multi-omics (CITE-seq) peripheral blood mononuclear cell (PBMC) dataset. ScLinear accurately predicts the protein levels irrespective of the individual protein (Fig. 2a). Notably, four known marker genes of the PBMC cell types (namely CD14, CD19, CD3, and CD56) clearly show the highest predicted abundance in the anticipated cell type (Fig. 2b). Similar performance was achieved in an additional human PBMC data set (Supplementary Fig. 1a, b), further supporting the confidence in scLinear’s modeling approach. Moreover, to investigate model robustness, the dropout rate in the two PBMC datasets was iteratively increased by randomly adding zeros to the gene expression matrices. The model performance was evaluated at each iteration and showed that the model predictions remain reliable even at very high dropout rates (Fig. 2c, Supplementary Fig. 1c).

Furthermore, to explore if this approach can be applied on data from other organisms, scLinear was trained and tested on CITE-seq data from mouse lymph node and spleen samples (Fig. 2d). One sample from the same tissue was used to train the scLinear model, while the second sample was used to test the trained model. We observe high correlation values for most of the proteins, similar to the human datasets. These results demonstrate a consistent performance, comparable to the human samples and show the generalizability of scLinear to other species. Taken together, scLinear performs at state-of-the-art levels, while being extremely more efficient and thus more scalable and widely applicable. Importantly, scLinear accurately generalizes in a zero-shot context, is robust at very high dropout rates and can be applied to other model organisms, as well.

### ScLinear’s generalizability to spatial data

One of the main limitations of scRNA-seq profiles is the lack of spatial context. Therefore, in recent years, spatial profiling technologies have emerged. Importantly, similar to single-cell multi-omics, it is now possible to simultaneously profile RNA and protein levels while maintaining the spatial context^12^. We, therefore, explored if scLinear and its pre-trained models are applicable in spatial cross modality prediction. We analyzed a dataset of Tonsil profiled with spatial transcriptomics and co-detection of 35 proteins.

**Fig. 3:**
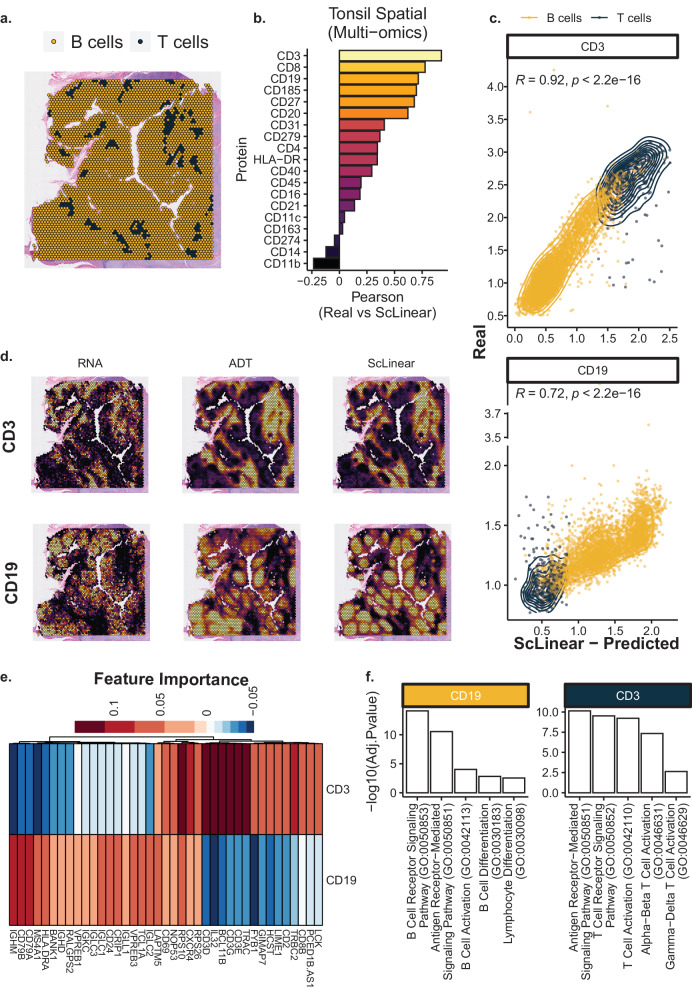
ScLinear application in spatial data and model interpretation. **a** Tonsil dataset analyzed by scLinear. Spots are colored by cell type. **b** Pearson correlation of 19 common proteins. **c** ADT vs predicted values for CD3 and CD19. **d** CD3 and CD19 RNA, protein and predicted levels. **e** Most significant features driving the prediction of CD19 and CD3. **f** Top 5 GO terms enriched in the predicted features shown in (**e**), split by protein. Two-sided Pearson correlation and one-tailed Fisher’s exact test were used. Fischer’s test pvalues are adjusted for multiple comparisons.

ScLinear’s cell type annotation revealed B cells to be the predominant cell type (Fig. 3a) consistent with what is expected^13^. 19 out of 35 proteins are common between the trained model and the Tonsil test dataset. Most of the proteins are accurately predicted with high Pearson correlation values (Fig. 3b), showcasing that although scLinear was trained in single-cell data, it can be successfully applied in spatial datasets. Similar to the PBMC data observation, CD3 and CD19 protein levels, the most known markers of T and B cells respectively, are remarkably predicted by scLinear in the relevant cell types (Fig. 3c). Interestingly, when exploring the different modalities and the predicted values, we clearly see that the predicted values better recapitulate the protein levels compared to the RNA (Fig. 3d), revealing the importance of such approaches even in spatial transcriptomics datasets where protein levels are not available. Finally, the undisputed and significant advantage of using a dimensionality reduction and linear regression-based approach, such as scLinear, is the straightforward interpretability of the model. We can directly calculate feature importance values that represent the influence of each input GEX feature on the predicted protein abundance. We therefore estimated the genes that drive the predictions of CD19 and CD3 in the Tonsil data as representative examples. We see that the top 20 features are highly different between the two proteins (Fig. 3e). Notably, CD3 genes (CD3E, D and G) are in the most important features for CD3 protein prediction. Gene Ontology (GO) analysis of the top features for CD3 and CD19 show enrichment in T and B cell related biological processes respectively (Fig. 3f), validating the biological relevance of scLinear’s models.

## Discussion

We present scLinear, a versatile tool which is based on a simple linear regression modeling approach and predicts protein levels from RNA expression. ScLinear is easily compatible with the most widely used scRNA-seq workflows and comes with pre-trained models making it broadly applicable. We show that scLinear performs at state-of-the-art levels while being vastly more efficient compared to more complex approaches. This fact makes scLinear more scalable and having a smaller carbon footprint. More importantly, we show that scLinear can generalize in unseen single-cell and spatial transcriptomics data, further showing its broad applicability. Finally, scLinear can be used to generate novel hypotheses by its straightforward interpretable nature.

Although we showed the performance and reliability of scLinear in protein abundance prediction, there are some limitations to account for, mostly arising from the availability and characteristics from the CITE-seq datasets. The measured proteins in the available CITE-seq datasets are, in their vast majority, immune-related. Therefore, although scLinear shows very accurate results in hematopoietic cells from healthy donors, its performance in pathological conditions and in modeling the abundance of non-immune protein markers remain to be explored.

We hypothesize that the superior performance of scLinear in this specific prediction task could be attributed to several reasons. Initially, our work focuses on predicting protein abundances from RNA levels, which may inherently be predictable with a linear model due to their direct biological relationship. In contrast, deep learning models, while highly flexible and powerful, might easily overfit to the training data, leading to poorer predictive performance on unseen data. Considering the bias-variance tradeoff, in instances where the available data size is relatively small or noisy, simpler models like linear regression can outperform training procedures with higher variance when evaluated on unseen data^14^. Even if we consider the double descent phenomenon, where deep neural networks have been shown to overcome the bias-variance tradeoff by achieving generalization in the overparametrized regime^15^, tuning their architecture and hyperparameters is much more challenging than fitting a linear regression on data preprocessed with established methods. Interestingly, similar observations have been done in other research fields where linear regression models outperformed neural networks or other more complex methodologies^16–18^.

Taken together, we show that, in some cases, simple, efficient, and interpretable algorithms such as linear regression are better suited even for challenging machine learning tasks. We propose that, in the future, such approaches should be explored in even more complex tasks, such as scATAC-seq modality prediction, against the trend of utilizing by default complicated ML/DL architectures.

## Methods

### Methods performance evaluation

Methods’ accuracy was evaluated by estimated the RMSE of the predictions, in concordance to the NeurIPS competition. We monitored the execution time and RAM usage of each method. All the methods were evaluated on the same hardware and software configuration (Laptop Intel i7 7th gen, Nvidia GTX 1060 mobile, 32 GB RAM, Ubuntu 22.04), and the monitoring of the system metrics over time was done using the experiment tracking library Wandb. It is important to note that the training time for the Babel_Dance and Vanilla_NN methods heavily depends on the GPU, since they are based on neural networks.

### ScLinear - Quality control, pre-processing and cell type annotation

ScLinear includes pre-processing functions designed to prepare a single cell RNA sequencing experiment for ADT prediction. These functions come with default settings that typically provide good quality control and pre-processing for most datasets. The Seurat framework is utilized to carry out these pre-processing steps^19^. First empty droplets can be removed using the DropletUtils package^20^. Next, the counts are log normalized, and the most variable features are identified and scaled with Seurat. Heterotypic doublets are identified and removed with the scDblFinder tool^21^. To evaluate the quality of the cells, three cell-based metrics are used, including the percent of mitochondrial reads, the amount of reads per cell and the amount of unique features detected per cell. Low quality outlier cells are determined based on the median absolute deviation (MAD) implementation from the scater package^22^ with a default threshold of 3 MAD. For the percent of mitochondrial reads, only the upper threshold is used, while for the other two metrics, the upper and lower thresholds are used. If multiple batches were sequenced, the data can be integrated with the anchor-based reciprocal PCA method from Seurat. To estimate the number of relevant principle components (PCs), the maxLikGlobalDimEst function from the intrinsicDimension package^23^ is used. Clustering of the data is carried out using the Louvain algorithm implemented in Seurat, and the resulting clusters are then annotated using the hierarchical human cell type marker set from PanglaoDB^24^ and the scMRMA package^25^.

### ScLinear - Protein abundance prediction

ScLinear’s ADT prediction pipeline is based on the best practices for single-cell protein abundance prediction from the winning methods of the NeurIPS 2021 Multimodal single cell data integration challenge. For the gene expression (GEX) data, we employ the same dimensionality reduction and normalization steps proposed by the winning team of the GEX2ADT modality prediction task (Guanlab – dengkw). Specifically, the first step involves fitting a truncated singular value decomposition (SVD) on the GEX matrix to reduce the feature dimensionality to 300 components. The second step consists in applying a cell-wise z-score normalization on the low-dimensional GEX matrix, this produces a matrix where each cell vector has a mean of 0 and a standard deviation of 1. After these transformations of the GEX matrix, we train a multivariate linear regression model to predict the ADT matrix.

We provide the ADT prediction pipelines pretrained on the B cells, NK cells, and T cells of the NeurIPS 2021 competition dataset, as well as the pipeline pretrained on the whole dataset. The dataset contains CITE-seq measurements of 66,175 bone marrow mononuclear cells (BMMCs) from different donors and collected in different sites. Note that the GEX data is normalized by dividing the UMI counts through the size factor calculated with scran and then log1p transformed. The ADT data is normalized using centered log ratio (CLR) transformation across cells, as implemented in Seurat. Therefore, the pretrained ADT prediction pipelines should be used on similarly normalized GEX data and the predicted ADT output should be interpreted as CLR-normalized.

scLinear’s pretrained ADT prediction pipelines can be applied to any GEX matrix. The function implemented in R takes as input a Seurat assay object. The gene names are used to compute the genes of the input GEX matrix which are in common with the genes on which the pipeline was trained on. The output of the pipeline consists of a Seurat assay object containing the predicted ADT matrix. The predicted ADT features are the same as the ones used in the training data.

### Feature importance

We express the feature importance as the Jacobian matrix of the predicted ADT with respect to the input GEX features. Since scLinear involves three components—truncated SVD, z-score normalization, and linear regression—we can decompose the Jacobian of the full model as the product of the Jacobians of these three components. For the truncated SVD, the Jacobian is simply the right singular vector matrix. For the linear regression, it is the weight matrix. For the z-score normalization, we compute the Jacobian using automatic differentiation provided by the PyTorch library. Thus, the resulting feature importance matrix contains the partial derivatives of each predicted protein with respect to each input gene. The feature importance values directly quantify the effect on the predicted ADT of perturbing each individual GEX feature. This enables straightforward interpretation of the most influential genes for predicting each protein. Functional enrichment analysis of the most informative genes was performed using Enrichr^26^ (version June 8, 2023) and the Gene Ontology Biological Processes gene set^27^ (2023 version). The pvalues are calculated with one-tailed Fisher’s exact test and are adjusted for multiple comparisons (Benjamini-Hochberg method).

### Dropout simulation

Dropout rate of the PBMC10K and PBMC5K data were iteratively increased to investigate prediction robustness. In each iteration, non-zero values from the count matrix were randomly sampled from a Bernoulli distribution with a probability of success of 10%. Subsequently, the sampled values were set to 0, increasing the dropout rate and the new dataset was used to predict the ADT values. This process was iteratively repeated until a dropout rate above 99% was reached. In each cell, the Pearson correlation between predicted and measured ADT values was calculated.

### Statistics and reproducibility

Correlation values are estimated with two-sided Pearson method. Mean values +/− the standard deviation are shown as barplots with error bars. Source data for the figures, ScLinear’s source code and a notebook to reproduce the figures are available in github (https://github.com/DanHanh/scLinear, https://github.com/DanHanh/scLinear_appendix), Zenodo (https://zenodo.org/records/10602787, https://zenodo.org/records/10602824) and in in Supplementary Data 1.

### Data processing

The PBMC data were loaded with R (v4.3.1) with Seurat (v4.4.0) and automatically processed by the prepare_data function from scLinear, as described under *“*ScLinear - Quality control, preprocessing and cell type annotation*”*. The following package versions were used: scDblFinder(v1.14.0), scater (v1.28.0), intrinsicDimension(v1.2.0), scMRMA (v1.0). Quality control on the ADT assay was performed with the cleanTagCounts function from DropletUtils(v1.20.0) with default parameters. After quality control, the isotype controls were removed from the ADT assay. The Tonsil data were processed using Seurat, spots containing more than 25% mitochondrial reads and less than 500 unique features were removed. Gene expression data was normalized with SCTransform, and the ADT assay using centered log ratio transformation as implemented in Seurat. The data was then clustered through the Louvain algorithm using the first 30 PCs. The predominant cell type for each spot was annotated with the scMRMA package.

### Reporting summary

Further information on research design is available in the Nature Portfolio Reporting Summary linked to this article.

### Supplementary information

Supplementary Information

Description of Additional Supplementary Files

Supplementary Data 1

Reporting summary

## Data Availability

The data used in this study are available in the public domain. The NeurIPS data are available in Gene Expression Omnibus (GSE194122). The PBMC, Tonsil, and Mouse data are available via the 10x Genomics public datasets webpage (https://www.10xgenomics.com/resources/datasets?query=&page=1&configure%5BhitsPerPage%5D=50&configure%5BmaxValuesPerFacet%5D=1000). PBMC10K: 10k PBMCs from a Healthy Donor - Gene Expression with a Panel of TotalSeq™-B Antibodies, Cell Ranger 3.0.0, 10x genomics, (2018, Nov., 19). PBMC5K: 5k Peripheral Blood Mononuclear Cells (PBMCs) from a Healthy Donor with a Panel of TotalSeq™-B Antibodies (v3 chemistry), Cell Ranger 3.1.0, 10X genomics (2019, July, 24). Tonsil: Visium CytAssist Gene and Protein Expression Library of Human Tonsil, H&E, 6.5 mm (FFPE), Space Ranger 2.1.0, 10x genomics, (2023, May, 15). Mouse: Mixture of Cells from Mouse Lymph Nodes and Spleen Stained with TotalSeq™-C Mouse Universal Cocktail, Cell Ranger 7.2.0, 10x genomics, (2023, Sep, 14). The source data behind Figures: 1b–d, 2a–d, 3b–c, 3e–f and Supplementary Figs.: 1a–c can be found in Supplementary Data 1.

## Code availability

The scLinear R package can be found at https://zenodo.org/records/10602787^28^ or at https://github.com/DanHanh/scLinear. Additional source code and accompanying information can be found at https://zenodo.org/records/10602824^29^ or at https://github.com/DanHanh/scLinear_appendix.
