# Supplementary material for: ScLinear predicts protein abundance at single-cell resolution
Source: Commun Biol. 2024 Mar 4;7:267. doi: 10.1038/s42003-024-05958-4 (PMC10912329; doi:10.1038/s42003-024-05958-4)
Supplement: Supplementary file 1 — Supplementary Information [file 42003_2024_5958_MOESM1_ESM.pdf]

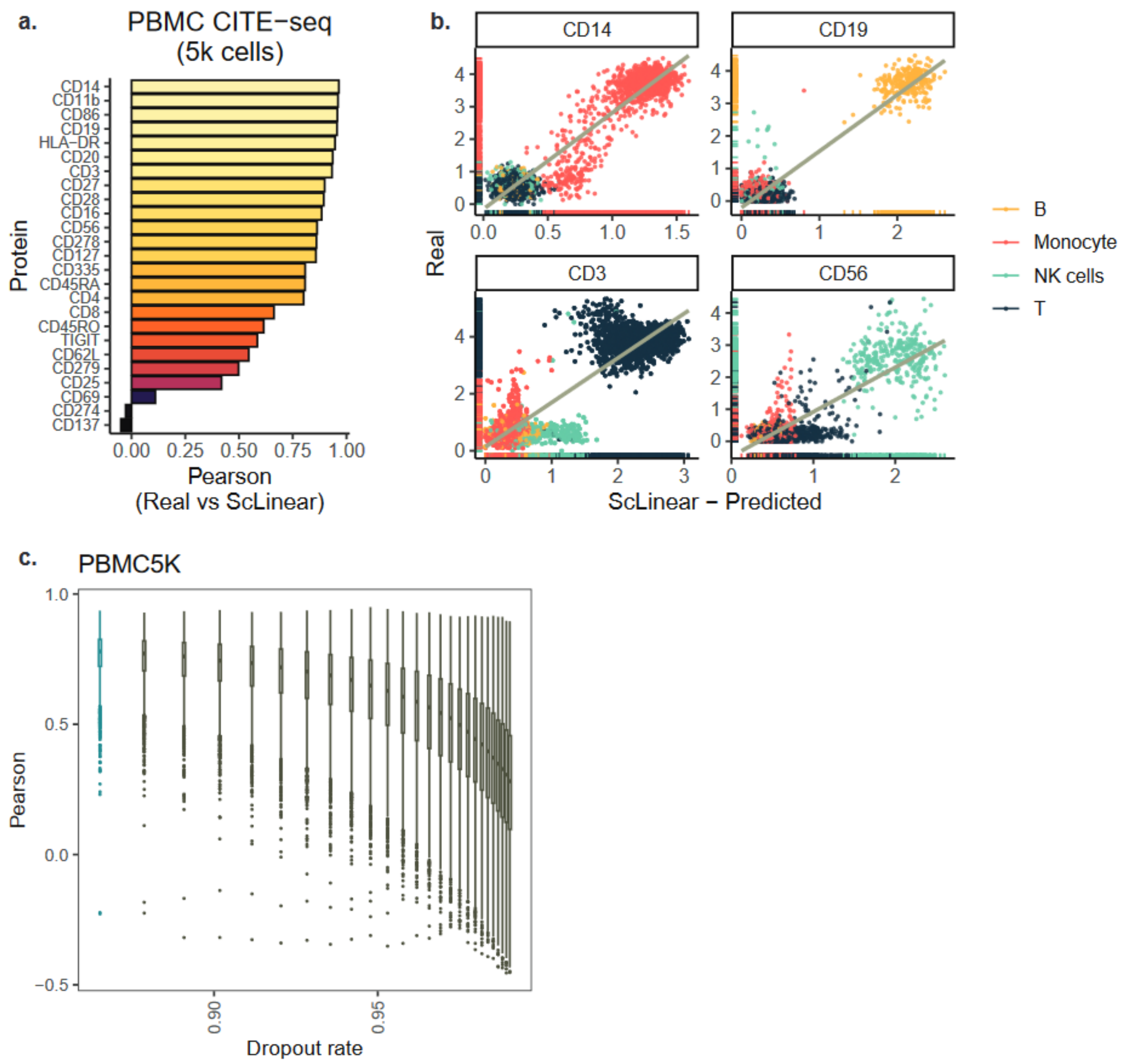

Supplementary Figure 1: ScLinear results for PBMC5K dataset. *a. Pearson Correlation in unseen PBMC5K data split by protein. b. ScLinear predictions in relationship to the measured data for 4 known makers colored by cell type. c. Pearson correlation of scLinear prediction values compared to ADT across different levels of dropouts. Two-sided Pearson correlation was used.*
