# Supplementary material for: ScLinear predicts protein abundance at single-cell resolution
Source: Commun Biol. 2024 Mar 4;7:267. doi: 10.1038/s42003-024-05958-4 (PMC10912329; doi:10.1038/s42003-024-05958-4)
Supplement: Supplementary file 2 — Description of Additional Supplementary Files [file 42003_2024_5958_MOESM2_ESM.pdf]

Description of Additional Supplementary Files

File name: Supplementary Data 1

Description: The source data behind the Figures: 1b-d, 2a -d, 3b-c, 3e-f and for Supplementary Figures: 1a-c
